# Dietary flaxseed: Cardiometabolic benefits and its role in promoting healthy aging

**DOI:** 10.1007/s11357-025-01512-0

**Published:** 2025-01-16

**Authors:** Setor K. Kunutsor, Davinder S. Jassal, Amir Ravandi, Andrea Lehoczki

**Affiliations:** 1 Section of Cardiology, Department of Internal Medicine, Rady Faculty of Health Sciences, Max Rady College of Medicine, University of Manitoba, St. Boniface Hospital, 409 Tache Avenue, Winnipeg, MB R2H 2A6 Canada; 2 Institute of Cardiovascular Sciences, Department of Physiology and Pathophysiology, Rady Faculty of Health Sciences, Max Rady College of Medicine, University of Manitoba, Winnipeg, MB Canada; 3 Institute of Preventive Medicine and Public Health, Semmelweis University, Budapest, Hungary; 4 Doctoral College, Health Sciences Program, Semmelweis University, Budapest, Hungary

**Keywords:** Flaxseed, Cardiometabolic, Type 2 diabetes, Hypertension, Metabolic syndrome, Chronic kidney disease, Cardiovascular disease, Mortality

## Abstract

Flaxseed, a rich source of omega-3 polyunsaturated fatty acid alpha-linolenic acid (ALA), lignans, and soluble fiber, has attracted attention for its potential to improve multiple cardiometabolic risk factors. While its benefits are well-recognized, comprehensive evaluations of its direct impact on clinical outcomes, such as the prevention or progression of cardiometabolic diseases, remain limited. Additionally, its potential to support healthy aging and longevity through fundamental biological mechanisms has not been fully elucidated. This review synthesizes existing research on flaxseed supplementation, highlighting its effects on cardiometabolic risk factors and outcomes, the underlying biological mechanisms, and its broader implications for health promotion and aging. Findings demonstrate that flaxseed supplementation significantly improves several cardiometabolic risk factors, including body weight, body mass index, lipid levels, blood pressure, glycemic measures, markers of inflammation (e.g., C-reactive protein and interleukin-6), oxidative stress, and liver enzymes. Blood pressure reductions range from approximately 2 to 15 mmHg for systolic blood pressure and 1 to 7 mmHg for diastolic blood pressure, with the magnitude influenced by dose, duration, and baseline risk profiles. While direct evidence linking flaxseed to the prevention of hypertension, metabolic syndrome, metabolic dysfunction-associated steatotic liver disease, type 2 diabetes, chronic kidney disease, and cardiovascular disease is limited, its bioactive components—ALA, lignans, and fiber—are strongly associated with reduced risks of these conditions. The benefits of flaxseed are mediated through multiple pathways, including anti-inflammatory and antioxidant effects, improved lipid levels, improved glucose metabolism and insulin sensitivity, modulation of gut microbiota, and enhanced vascular health. Beyond cardiometabolic outcomes, flaxseed may influence key biological processes relevant to aging, underscoring its potential to promote healthy aging and longevity. Optimal cardiometabolic benefits appear to be achieved with ground whole flaxseed at doses of ≥ 30 g/day for at least 12 weeks, particularly among individuals at high cardiometabolic risk. Future research should focus on elucidating flaxseed’s mechanisms of action, clarifying its role in disease prevention, and refining dietary recommendations to harness its potential for cardiometabolic health and aging interventions.

## Introduction

Unhealthy aging is a growing global concern, characterized by the accelerated decline in physiological and metabolic functions, leading to increased susceptibility to age-related diseases. Among these are cardiometabolic diseases, including hypertension, metabolic syndrome (MetS), metabolic dysfunction-associated steatotic liver disease (MASLD), type 2 diabetes (T2D), chronic kidney disease (CKD), and cardiovascular diseases (CVDs), which collectively represent a significant public health burden. As populations worldwide continue to age, the prevalence of these conditions is projected to rise, placing additional strain on healthcare systems and diminishing quality-adjusted life years.

The aging process, particularly unhealthy aging, is intricately linked to chronic, low-grade inflammation, oxidative stress, and metabolic dysregulation, which collectively drive the development of cardiometabolic diseases. Hypertension, affecting over a billion people globally, is a leading cause of premature death, with untreated high blood pressure contributing to severe vascular complications such as coronary heart disease (CHD) and stroke. Its burden is disproportionately high in low- and middle-income countries [1,2]. MetS, a cluster of conditions including elevated blood pressure, insulin resistance, abdominal obesity, and dyslipidemia, is increasingly prevalent due to the global rise in obesity and sedentary.

lifestyles [3–6].

MASLD, formerly known as nonalcoholic fatty liver disease (NAFLD), further exemplifies the interplay between aging, obesity, and metabolic dysfunction. This condition, characterized by fat accumulation in the liver independent of alcohol consumption, often coexists with obesity, MetS, and T2D, significantly increasing the risk of liver cirrhosis and CVD [7]. Similarly, T2D, marked by insulin resistance and chronic hyperglycemia, is a critical cardiovascular risk factor and is a major driver of healthcare costs and morbidity [8,9]. CKD, often a consequence of hypertension or T2D, affects approximately 10% of the global population, frequently leading to cardiovascular complications and kidney failure [10–12]. CVDs, encompassing conditions such as CHD, stroke, and heart failure, remain the leading cause of death globally, responsible for nearly 18 million deaths annually [13,14].

Unhealthy aging is exacerbated by lifestyle factors such as tobacco use, physical inactivity, excessive alcohol consumption, and poor dietary habits. Addressing these modifiable factors offers significant potential for mitigating the burden of age-related diseases and promoting healthier aging trajectories.

Among lifestyle interventions, dietary strategies have demonstrated substantial efficacy in improving cardiometabolic health and reducing disease risk. Diets such as the Mediterranean diet, which emphasize fruits, vegetables, whole grains, and healthy fats, have consistently been associated with reduced CVD risk and improved cardiometabolic profiles [15–23]. In recent years, attention has shifted toward functional foods—those providing health benefits beyond basic nutrition—for their potential role in promoting healthy aging. Functional foods rich in bioactive compounds, including n-3 polyunsaturated fatty acids (PUFAs), have shown promise in preventing cardiometabolic diseases [24–27].

Flaxseed, a plant-based source of the n-3 PUFA alpha-linolenic acid (ALA), lignans, and dietary fiber, is gaining recognition as a functional food with the potential to address the challenges of unhealthy aging [24]. Historically valued for its nutritional and medicinal properties, flaxseed has emerged as a focus of research for its ability to improve blood pressure, lipid profiles, glucose metabolism, and other cardiometabolic outcomes [24,28–30]. Its bioactive components not only target cardiometabolic risk factors but also modulate fundamental biological processes, such as inflammation and oxidative stress, that are central to aging.

Despite promising evidence, the role of flaxseed in promoting healthy aging and reducing the risk of age-related diseases requires further exploration. This review aims to synthesize existing research on the effects of flaxseed supplementation on cardiometabolic risk factors and outcomes, elucidate its mechanisms of action, and evaluate its potential in supporting longevity and resilience against age-related diseases. By addressing gaps in the evidence and discussing clinical and policy implications, this review seeks to guide future research and inform dietary recommendations for promoting cardiometabolic health and healthy aging.

## Flaxseed and its bioactive components

Flaxseed (*Linum usitatissimum*), commonly referred to as linseed in industrial applications, is one of the oldest cultivated crops, historically valued for its medicinal, nutritional, and industrial uses. Flax is a flowering plant with delicate blue or purple blossoms that produce fruit capsules, each containing up to 10 seeds. These seeds, known as flaxseeds, are golden or brown in color and rich in oils [31]. Traditionally, flaxseed oil is extracted by pressing or grinding the seeds, prized for its high omega-3 content. Flaxseeds can also be consumed whole or ground, offering numerous health benefits. Flaxseed refers to the plant when consumed for human health, while linseed is used in industrial contexts. Flax is cultivated globally, with significant production concentrated in countries such as Canada, Russia, China, the United States, and Kazakhstan. Canada is the world’s largest producer of flaxseed, accounting for a substantial portion of the global supply, and exports primarily to countries in Asia and Europe. Flax cultivation is valued not only for its seeds, which are used for nutritional and industrial purposes, but also for its fiber, which is integral to textile industries.

Flaxseed is nutritionally dense, containing several bioactive components that contribute to its extensive health-promoting properties. Key components include alpha-linolenic acid (ALA), lignans, dietary fiber, proteins and peptides, and cyanogenic glycosides [32]. ALA, an omega-3 PUFA, makes up 50–60% of flaxseed oil and about 22% of whole flaxseed [33]. Flaxseed is the richest plant source of omega-3 ALA, which cannot be endogenously synthesized. ALA plays a crucial role in human health as a precursor to longer-chain omega-3 fatty acids, eicosapentaenoic acid (EPA), and docosahexaenoic acid (DHA), which are found in marine-based sources and are vital for cardiovascular and neurological health [34]. While ALA is metabolized into EPA and DHA in the body, this conversion is limited, often competing with omega-6 linoleic acid (LA) [34], a PUFA that cannot be endogenously synthesized and predominates in Western diets. Maintaining an optimal omega-6:omega-3 ratio is critical to supporting anti-inflammatory processes and mitigating chronic disease risks. With its high ALA content, flaxseed serves as a functional food to rebalance this ratio, delivering cardiovascular and anti-inflammatory benefits. Flaxseed also contains lignans, with secoisolariciresinol diglucoside (SDG) being the most predominant lignan and making about 1% of dry weight. After ingestion, SDG is converted to secoisolariciresinol, which is further metabolized to the mammalian lignans enterodiol and enterolactone by bacteria [35,36]. Flaxseed SDG and its metabolites possess potent antioxidant and estrogenic properties [37]. Flaxseeds are rich in dietary fiber, comprising approximately 25% soluble fiber and 75% insoluble fiber [33]. The soluble fiber promotes gut health, aids in cholesterol reduction, and supports glycemic control [38]. Flax proteins and bioactive peptides, including linusorbs, exhibit antihypertensive and antioxidant properties. Flaxseed also contains cyanogenic glycosides, such as linustatin and neolinustatin, which release small amounts of hydrogen cyanide when metabolized [39]. However, these levels are generally safe for human consumption and further minimized through processing.

Flaxseed is available in various forms: whole flaxseed, ground or milled flaxseed (flax meal), flaxseed oil, and partially defatted flaxseed meal [24]. They vary in their nutritional composition with each offering distinct health benefits (Table 1). Whole flaxseed provides fiber and lignans but requires grinding to release ALA. Whole flaxseeds have a longer shelf life but may pass through the digestive system largely undigested. Ground flaxseed is devoid of the outer shell, making ALA and other nutrients more accessible. Ground flaxseed offers a balanced nutritional profile of ALA, lignans, and fiber [40,41]. Flaxseed oil is high in ALA, but lacks the fiber and lignans found in whole or ground seeds. It is sensitive to heat and light, so refrigeration is recommended to preserve its potency. Partially defatted flaxseed meal, a by-product of oil extraction, retains significant amounts of protein, fiber, and lignans, making it a valuable nutritional option.

**Table 1:** Nutritional compositions of the four main forms of flaxseed

Form of flaxseed	Nutritional composition
Whole Flaxseed	- High in dietary fiber (25–28% by weight) with both soluble and insoluble fiber - Rich in lignans, a type of phytoestrogen - Contains ~ 41% fat, primarily omega-3 ALA (~ 50–60% of total fat) - ~ 20% protein content - Requires grinding for optimal nutrient bioavailability
Ground or Milled Flaxseed	- Same nutritional composition as whole flaxseed but more bioavailable - High in fiber (~ 28%), with soluble fiber aiding gut health and cholesterol reduction - Rich in ALA (~ 50–60% of total fat content) - ~ 20% protein content - Lignans and fiber remain intact for health benefits
Flaxseed Oil	- Concentrated source of omega-3 ALA (~ 55–60%) - Contains negligible fiber and lignans - Minimal protein content - Sensitive to heat and light; refrigeration recommended
Partially Defatted Flaxseed Meal	- Lower fat content (~ 10–12%) due to oil extraction - High in protein (~ 30–35%) - Retains fiber (~ 28%) and lignans - Suitable for low-fat diets while retaining fiber and lignan benefits

## Methods

This review involved a comprehensive search of MEDLINE and Embase databases up to December 2024, focusing on randomized controlled trials (RCTs), non-RCTs, and observational research such as prospective cohort, nested case–control, case-cohort, and retrospective cohort studies. Emphasis was placed on systematic reviews and meta-analyses, based on the hierarchy of evidence [42]. Keywords used in the search included terms for flaxseed (“flax,” “flaxseed,” “linseed”) as well as terms related to various cardiometabolic risk factors and outcomes (e.g., “blood pressure,” “lipids,” “glucose,” “inflammation,” “hypertension,” “metabolic syndrome,” “MASLD”, “NAFLD,” “type 2 diabetes,” “chronic kidney disease,” “cardiovascular disease,” “coronary heart disease,” “stroke,” “heart failure,” “atrial fibrillation,” “mortality”). The review was limited to human studies published in English. Observational studies were prioritized if they were longitudinal cohort studies, which are well-suited for examining temporal relationships. For RCTs, we only included studies that directly or indirectly evaluated the effects of flaxseed supplementation alone compared with other interventions. Studies that specifically reported the effect of a combination of flaxseed supplementation and an additional intervention such as other dietary supplements, were not included. We extracted and reported risk estimates (mean differences and standardized mean differences (SMDs)) for associations demonstrating significant effects. This review did not specifically focus on the individual impact of the key bioactive components (ALA, lignans or fibre) on cardiometabolic risk factors and outcomes, as these relationships have been extensively investigated, with evidence showing they each exert beneficial cardiometabolic effects on their own.

## Impact of flaxseed supplementation on cardiometabolic risk factors and outcomes

### Cardiometabolic risk factors

Dietary flaxseed supplementation offers significant benefits across a wide range of cardiometabolic risk factors, making it a valuable addition to dietary strategies for improving overall metabolic health. Regular consumption of flaxseed has been shown to positively impact body weight and body mass index (BMI), supporting healthy weight management and reducing the risk of obesity-related complications.

Flaxseed’s lipid-lowering properties are well-documented, with consistent evidence of reductions in total cholesterol (TC), low-density lipoprotein cholesterol (LDL-C), and triglycerides (TGs). These effects, however, may vary depending on the form and type of flaxseed consumed, with ground flaxseed and flaxseed oil showing differing impacts on specific lipid parameters. Additionally, flaxseed exhibits potent anti-inflammatory and antioxidant effects, as evidenced by reductions in key biomarkers such as high sensitivity C-reactive protein (hsCRP), tumor necrosis factor-alpha (TNF-α), interleukin-6 (IL-6), and malondialdehyde (MDA). These properties highlight flaxseed’s role in mitigating chronic low-grade inflammation and oxidative stress, which are critical drivers of cardiometabolic diseases.

Flaxseed also improves glycemic control by enhancing insulin sensitivity and stabilizing blood glucose levels, making it particularly beneficial for individuals at risk of or managing T2D. Its high fiber content slows glucose absorption, while its lignans and ALA contribute to improved metabolic profiles.

One of flaxseed’s most pronounced effects is its ability to lower blood pressure. Studies consistently report significant reductions in both systolic blood pressure (SBP) and diastolic blood pressure (DBP), with greater effects observed in individuals with elevated baseline blood pressure or those receiving higher doses of flaxseed for longer durations.

In addition, flaxseed supplementation has demonstrated beneficial effects on liver health, as reflected in reduced levels of key liver markers such as alanine aminotransferase (ALT), aspartate aminotransferase (AST), and gamma-glutamyltransferase (GGT). These findings suggest that flaxseed may play a role in mitigating liver inflammation and injury, particularly in conditions such as MASLD. Collectively, these findings underscore the broad cardiometabolic benefits of flaxseed, which extend beyond individual risk factors to address systemic processes such as inflammation, oxidative stress, and metabolic dysregulation. Its diverse bioactive components make flaxseed a functional food with significant potential to improve cardiometabolic health and reduce the burden of related diseases.

#### Anthropometric indices

Mohammadi-Sartang [43] and colleagues in 2017 pooled the results of 45 RCTs (with 49 treatment arms) and showed a significant reduction in body weight (0.99 kg), BMI (0.30 kg/m^2^), and waist circumference (WC) (0.80 cm) following flaxseed supplementation at doses ranging from 13–90 g/day for 3–48 weeks. Subgroup analyses indicated that the impact of flaxseed supplementation on anthropometrics indices were more pronounced for whole flaxseed in doses ≥ 30 g/day, longer-term supplementation (≥ 12 weeks) and studies including participants with higher BMI (≥ 27 kg/m^2^) [43]. In a 2021 systematic review and dose–response meta-analysis involving 31 RCTs, Yang and colleagues [44] showed no significant effect of flaxseed supplementation (duration of 2–12 months) on body weight, BMI, WC or waist-to-hip ratio (WHR) in patients with dyslipidemia-related diseases. In subgroup analysis, (i) whole flaxseed supplementation significantly reduced body weight (0.40 kg); (ii) flaxseed oil significantly reduced WC (1.61 cm); and (iii) whole flaxseed increased WHR (0.02) [44]. In 2022, Jiang and colleagues [45] conducted a retrospective cohort study in which the relative efficacy of flaxseed oil and fish oil on the cardiovascular health of patients with T2D and CHD was evaluated. The flaxseed oil group received 1,000 mg flaxseed oil (containing 400 mg of α-linolenic acid) and the fish oil group received 1,000 mg of fish oil (containing 250 mg of EPA and 150 mg of DHA). After a median follow-up time of 10 weeks, there were no significant differences in body weight or BMI between the flaxseed oil and fish oil groups [45]. Xi and colleagues [46] in 2023 conducted a systematic review and meta-analysis of 13 RCTs to evaluate the impact of flaxseed supplementation on cardiometabolic parameters in patients with T2D. Flaxseed supplementation at doses ranging from 10–30 g/day for 8–25.7 weeks had no significant effect on body weight or BMI [46]. In pooled analysis of 5 RCTs of flaxseed oil supplementation at doses ranging from 2–40 g/day for 2–4 months, Tabrizi and colleagues [47] in 2024 showed no significant effect of flaxseed oil supplementation on body weight and BMI in patients undergoing hemodialysis. In a 2024 systematic review and meta-analysis involving 6 RCTs, Sabet and colleagues [29] showed no significant differences in either body weight or BMI following flaxseed intake ranging from 10–24 weeks in patients with coronary artery disease (CAD). In pooled analysis of 64 trials, Musazadeh and colleagues [48] in 2024 reported significant reductions in body weight (0.63 kg), BMI (0.24 kg/m^2^), and WC (1.43 cm) following flaxseed supplementation at doses ranging from 10–100 g/day for 2–24 weeks. Subgroup analyses indicated that the impact of flaxseed on anthropometrics indices was more pronounced for interventions lasting 10–20 weeks, and studies involving participants with BMI > 30 kg/m^2^ [48].

Flaxseed supplementation is associated with modest reductions in anthropometric indices such as body weight, BMI, and WC, though results vary across studies. More pronounced effects are observed with whole flaxseed supplementation at doses of ≥ 30 g/day, longer intervention durations (≥ 12 weeks), and among participants with higher BMI (≥ 27 kg/m^2^ or > 30 kg/m^2^). While some studies reported no significant changes, pooled analyses indicate that flaxseed supplementation is most effective for weight and WC reduction in targeted subgroups and with consistent, long-term use.

#### Lipids

In 2009, Pan and colleagues [49] pooled the results of 28 RCTs published between 1990 and 2008 to quantify the effectiveness of flaxseed and its derivatives on blood lipid profiles. Flaxseed supplementation at doses ranging from 20–50 g/day for 2–52 weeks significantly reduced TC (0.10 mmol/L) and LDL-C (0.08 mmol/L), with no effect on high-density lipoprotein cholesterol (HDL-C) and TGs. In subgroup analysis, (i) reductions in TC and LDL-C were significant for whole flaxseed and lignan supplements; (ii) reductions in TC and LDL-C were greater in women than men; and (iii) reductions in TC and LDL-C were significant for studies including participants with high initial concentrations of these lipids [49]. In 2012, Health Canada’s Food Directorate conducted a meta-analysis to evaluate a therapeutic claim regarding the cholesterol-lowering effects of ground flaxseed [50]. The analysis included 8 clinical trials involving normocholesterolemic and hypercholesterolemic men and women, with flaxseed supplementation doses ranging from 30 to 50 g/day over durations of 4 weeks to 12 months. All but one study used ground flaxseed. The findings revealed significant reductions in TC by 0.21 mmol/L and LDL-C by 0.22 mmol/L. Based on these results, Health Canada’s Food Directorate concluded that there is sufficient scientific evidence to support the claim that ground whole flaxseed lowers blood cholesterol levels [50]. In a 2020 systematic review and dose–response meta-analysis of 62 RCTs, Hadi and colleagues [51] showed that flaxseed supplementation at doses ranging from 10–60 g/day for 2–54 weeks significantly reduced levels of TC (5.39 mg/dL), TGs (9.42 mg/dL), and LDL-C (4.21 mg/dL), with no effects on HDL-C. In subgroup analysis, (i) LDL-C was significantly reduced in trials conducted in unhealthy participants with high baseline LDL-C, or normal and overweight participants, postmenopausal and participants with dyslipidemia, and in trials that administered whole flaxseed and lignin supplement for > 12 weeks and (ii) TGs decreased following flaxseed supplementation in trials that administered whole flaxseed, for either a short or long duration, as well as studies that included individuals with baseline BMI ≥ 25 kg/m^2^, unhealthy participants, or those with abnormal TG levels and patients with polycystic ovarian syndrome [51]. In another 2020 meta-analysis involving 7 RCTs, Hadi and colleagues [52] showed that flaxseed supplementation at doses ranging from 30–50 g/day for 3–54 weeks significantly reduced levels of lipoprotein[a] (Lp[a]) (2.06 mg/dl). Subgroup analysis also revealed that the significant lowering effect of flaxseed supplementation on Lp[a] was driven by longer duration of treatment (≥ 12 weeks). Sahebkar and colleagues [53] in 2021 pooled the results of 6 RCTs and showed a significant decrease in Lp[a] levels (SMD: −0.22) following supplementation with flaxseed-containing products. In a 2021 systematic review and dose–response meta-analysis involving 31 RCTs, Yang and colleagues [44] showed significant reductions in TC (8.73 mg/dL), LDL-C (6.92 mg/dL), TGs (12.31 mg/dL), Apo A (4.14 mg/dL), and Apo B (4.70 mg/dL), with no significant change for HDL-C, TC/HDL-C ratio, and Apo B/Apo A ratio following flaxseed supplementation (duration of 2–12 months). In subgroup analysis, (i) the reductions in TC and LDL-C were significant for whole flaxseed and lignan supplementation but not flaxseed oil; (ii) the reduction in TGs was driven by whole flaxseed supplementation; and (iii) the reductions in Apo A and Apo B were driven by whole flaxseed supplementation [44]. In a 2022 systematic review and meta-analysis of 14 RCTs, Masjedi and colleagues [54] evaluated the effect of flaxseed supplementation at doses ranging from 2–40 g/day for 1–12 weeks on the lipid profiles of healthy compared with patients with dyslipidemia. In healthy participants, flaxseed supplementation significantly reduced levels of TC (SMD: −16.53) and increased levels of LDL-C (SMD: 2.41) and HDL-C (SMD: 5.12), with no effect on TGs. In the subgroup analysis of healthy participants: (i) the reduction in TC was significant for participants with BMI > 25 kg/m^2^; (ii) the increase in HDL-C was significant for low dose flaxseed, BMI > 25 kg/m^2^, and flaxseed oil; (iii) LDL-C was decreased for high dose flaxseed, BMI > 25 kg/m^2^, and non-oil flaxseed [54]. In patients with dyslipidemia, flaxseed supplementation significantly reduced levels of TC (SMD: −1.41), LDL-C (SMD: −0.69), and TGs (SMD: −1.47), with no effect on HDL-C. In the subgroup analysis, (i) treatment duration < 8 weeks and non-oil flaxseed significantly increased HDL-C and (ii) treatment duration > 8 weeks and flaxseed oil supplementation significantly reduced HDL-C levels [54]. In the 2023 meta-analysis by Xi and colleagues [46], flaxseed supplementation at doses ranging from 10–30 g/day for 8–25.7 weeks had no significant effects on TC, TGs, HDL-C, or LDL-C. In subgroup analyses, (i) there was a significant reduction in TC when participants were < 60 years old, at normal weight, and supplemented with whole flaxseed or flax gum and (ii) whole flaxseed supplementation significantly increased HDL-C and reduced LDL-C levels in participants with T2D [46]. Benam and colleagues [55] in 2024 evaluated the effects of flaxseed supplementation on lipoproteins in a systematic review and meta-analysis of 18 RCTs. In their pooled analysis, flaxseed supplementation at doses ranging from 360–50,000 mg/day for 3–48 weeks significantly reduced levels of apo-BI (SMD: − 0.57) and lipoprotein (a) (SMD: − 0.34), with no effect on apo-AI levels [55]. In subgroup analysis, (i) flaxseed oil supplementation was associated with a decrease in apo-AI in patients with hypertension, in those with a BMI < 27 kg/m^2^ and a mean age > 50 years and (ii) the effect of flaxseed supplementation on apo-BI levels was greater in patients with hyperlipidemia, with a mean age ≤ 50 years and an intervention duration of < 12 weeks, and in trials based on whole flaxseed [55]. In pooled analysis of 5 RCTs, Tabrizi and colleagues [47] in 2024 showed a significant decrease in levels of TGs (85.78 mg/dL), with no effect on HDL-C, LDL-C and TC, following flaxseed oil supplementation at doses ranging from 2–40 g/day for 2–4 months in patients undergoing hemodialysis. Ahmed and Fateh [56] in 2024 pooled the results of 5 RCTs conducted in patients with NAFLD and showed significant reductions in TGs (230.72 mg/dl), TC (216.25 mg/dl), and LDL-C (210.67 mg/dl) and an increase in HDL-C (1.82 mg/dl) following flaxseed supplementation for 8–12 weeks. In a 2024 systematic review and meta-analysis involving 6 RCTs, Sabet and colleagues [29] showed no significant differences in either TGs, TC, LDL-C or HDL-C following flaxseed intake lasting 10–24 weeks in patients with CAD.

Flaxseed supplementation is associated with significant reductions in TC, LDL-C, TGs, and Lp[a], with no consistent effects on HDL-C. These benefits are more pronounced with ground whole flaxseed supplements at doses of ≥ 30 g/day, intervention durations of ≥ 12 weeks, and among subgroups such as individuals with dyslipidemia, higher BMI (> 25 kg/m^2^), and postmenopausal women. Subgroup analyses also highlight greater reductions in lipid levels in participants with high baseline lipid concentrations and specific populations like patients with NAFLD or hyperlipidemia.

#### Markers of inflammation, oxidative stress and endothelial function

In 2014, Caligiuri and colleagues conducted a study to assess the effects of flaxseed consumption on pro-inflammatory oxylipin levels in healthy adults from two age groups: younger adults (19–28 years) and older adults (45–64 years). At baseline, the older group showed significantly higher levels of several pro-inflammatory oxylipins compared to the younger group. Following a four-week intervention of consuming muffins containing 30 g of milled flaxseed daily, oxylipin levels in the older group significantly decreased, aligning with levels observed in the younger participants [57]. Mirfatahi and colleagues [58] in 2016 using a RCT investigated the effects of flaxseed oil consumption on serum systemic and vascular inflammation markers, and oxidative stress in hemodialysis patients. The patients in the flaxseed oil group received 6 g/day flaxseed oil for 8 weeks, whereas the control group received 6 g/day medium-chain triglycerides (MCT) oil. At the end of week 8, the flaxseed oil group showed significant reductions in hsCRP and soluble vascular cell adhesion molecule type 1 (sVCAM-1), with no significant differences in soluble intercellular adhesion molecule type 1 (sICAM-1), sE-selectin, and MDA [58]. Ren and colleagues [59] in a 2016 meta-analysis of 20 RCTs showed that flaxseed supplementation at doses ranging from 13–60 g/day for 2–52 weeks had no significant effect on CRP levels. However, meta-regression analysis revealed a significant reduction in CRP levels (0.83 mg/L) among participants with a BMI of ≥ 30 kg/m^2^ ^59^. Ursoniu and colleagues [60] in a 2019 meta-analysis of 17 RCTs showed no significant effect of flaxseed supplementation at doses ranging from 13–60 g/day for 2–52 weeks, on CRP levels. Subgroup analysis did not show evidence of effect modification by type of flaxseed supplement [60]. Rahimlou and colleagues [61] in a 2019 meta-analysis involving 32 RCTs showed that flaxseed supplementation at doses ranging from 360 mg-60 g/day for 2–12 weeks significantly reduced hsCRP (0.75) and TNF-α (0.38), with no effect on IL-6 and CRP. The findings did not vary significantly by supplement type, age group, BMI, study duration, and participants’ gender [61]. In a 2020 systematic review and meta-analysis of 40 RCTs, Askarpour and colleagues [62] evaluated the efficacy of flaxseed supplementation on major adhesion molecules and inflammatory cytokines in adults. Their results showed that flaxseed supplementation at doses ranging from 13–60 g/day for 2–54 weeks, reduced the concentrations of CRP (0.387 mg/L), IL-6 (0.154 pg/Ml), and VCAM-1 (22.809 ng/ml), with no significant effect on TNF-α, ICAM-1, and E-selectin. In 2020, Tamtaji and colleagues [63] conducted a meta-analysis to evaluate the effects of flaxseed oil supplementation on biomarkers of inflammation and oxidative stress in patients with MetS and related disorders. In pooled analysis of 12 RCTs, flaxseed supplementation with durations ranging from 4 weeks to 120 days resulted in a significant reduction in IL-6 (0.22) and MDA (0.17) and a significant increase in total antioxidant capacity (TAC) levels (137.25), with no effect on CRP, TNF-α, nitric oxide (NO), and glutathione (GSH) [63]. In a 2021 systematic review and dose–response meta-analysis involving 31 RCTs, Yang and colleagues [44] showed a significant reduction in IL-6 levels (0.23 pg/mL), with no significant changes for CRP and TNF-α following flaxseed supplementation (duration of 2–12 months). In subgroup analysis, (i) the reduction in IL-6 was driven by flaxseed oil but not whole flaxseed supplementation and (ii) flaxseed oil reduced levels of hsCRP [44]. Musazadeh and colleagues [64] in 2021 pooled the results of 8 RCTs and showed that flaxseed oil supplementation for 4–12 weeks led to a significant decrease in MDA levels (SMD: −0.52) and an increase in TAC levels (82.84 mmol/L), with no significant effect on GSH. In the 2022 retrospective cohort analysis by Jiang and colleagues [45], flaxseed oil significantly reduced hsCRP levels compared to fish oil. In pooled analysis of 5 RCTs, Tabrizi and colleagues [47] in 2024 showed a significant decrease in CRP levels (2.66 mg/L) following flaxseed oil supplementation at doses ranging from 2–40 g/day for 2–4 months in patients undergoing hemodialysis. In a 2024 systematic review and meta-analysis involving 6 RCTs, Sabet and colleagues [29] showed a significant reduction in hsCRP levels (1.35 md/L) following flaxseed intake ranging from 10–24 weeks in patients with CAD. In a systematic review and meta-analysis of 54 RCTs, Musazadeh and colleagues [65] in 2024 showed that flaxseed supplementation at doses ranging from 13–100 g/day for 2–52 weeks significantly reduced CRP (SMD: −0.46), and IL-6 (SMD: −0.64), with no effect on TNF-α. In subgroup analysis, (i) CRP was significantly reduced in trials conducted in hemodialysis patients and those with a mean age < 50 years and high-quality trials administering whole flaxseed supplementation, particularly in trials lasting < 12 weeks and (ii) the effect on IL-6 levels was significant in high-quality trials that used whole flaxseed supplementation for durations of ≥ 12 weeks, as well as in studies involving hyperlipidemic patients, particularly those under the age of 50 years [65].

Flaxseed supplementation significantly reduces markers of inflammation such as hsCRP, IL-6, and TNF-α, oxidative stress markers like MDA, and improves TAC, with no consistent effects on other markers like E-selectin or GSH. These effects are more pronounced with flaxseed oil at doses ≥ 13 g/day, interventions lasting ≥ 12 weeks, and in subgroups including hemodialysis patients, individuals with BMI ≥ 30 kg/m^2^, and hyperlipidemic patients. Improvements in endothelial function markers such as VCAM-1 are also observed, particularly with longer supplementation durations.

#### Measures of glycemia

Mohammadi-Sartang and colleagues [66] in 2018 pooled the results of 25 RCTs (30 treatment arms) and showed that flaxseed supplementation for 2–48 weeks significantly reduced blood glucose (2.94 mg/dL), insulin levels (7.32 pmol/L), and Homeostatic Model Assessment for Insulin Resistance (HOMA-IR) index (0.49), and increased quantitative insulin sensitivity check index (QUIKI) (0.019); there was no significant effect on HbA1c [66]. In subgroup analyses, these beneficial effects were driven by studies that used whole flaxseed but not flaxseed oil and lignan extract; furthermore, significant reductions were observed for insulin levels and insulin sensitivity indices in trials that lasted ≥ 12 weeks [66]. Villarreal-Renteria and colleagues [67] in 2022 pooled the results of 7 RCTs studies and showed a significant reduction in fasting blood glucose (FBG) (SMD: −0.392), insulin concentrations, (SMD: −0.287), HbA1c (SMD: −0.442), and HOMA-IR (SMD: −0.284) following flaxseed supplementation at doses ranging from 13–40 g/day for 8–12 weeks. In the 2022 retrospective cohort analysis by Jiang and colleagues [45], flaxseed oil compared with fish oil significantly reduced serum insulin levels, with no effect on HOMA-IR and FBG. In the 2023 meta-analysis by Xi and colleagues [46], flaxseed supplementation at doses ranging from 10–30 g/day for 8–25.7 weeks significantly reduced HbA1c (0.19%), but had no effects on FBG, HOMA-IR index, and QUIKI. In subgroup analyses, (i) patients < 60 years experienced a significant decrease in insulin levels after flaxseed supplementation; (ii) flaxseed supplementation significantly decreased FBG in normal weight participants (BMI: 18.5–24.9 kg/m2) and insulin concentrations in overweight participants (BMI: 25–29.9 kg/m2), (iii) FBG was significantly reduced with flaxseed supplementation in participants with baseline FBG ≥ 8.0 mmol/L or baseline HbA1c ≥ 7.0%, and (iv) there was a significant decrease in HbA1c in participants with baseline HbA1c ≥ 7.0% [46]. In 2023, Kavyani and colleagues [68] evaluated the effect of flaxseed supplementation on glycemic control in a systematic review and meta-analysis of 47 RCTs and showed that flaxseed supplementation at doses ranging from 1000–100,000 mg/day for 2–52 weeks**,** significantly reduced FBG (SMD: − 0.66), HOMA-IR (SMD: − 0.64), and insulin (SMD: − 0.49), with an increase in QUICKI (SMD: 1.64); however, flaxseed supplementation had no significant effect on HbA1c. In subgroup analyses, (i) whole flaxseed supplementation significantly reduced FBG, with the greatest effect in patients with T2D, duration of intervention < 12 weeks, and average age ≤ 50 years, (ii) whole flaxseed significantly reduced HbA1c levels in patients with T2D with a baseline BMI of ≥ 30 kg/m^2^ and an intervention duration of ≥ 12 weeks, (iii) whole flaxseed supplementation significantly reduced HOMA-IR, with the greatest effect for intervention duration of ≥ 12 weeks, mean age ≤ 50 years, and in patients with NAFLD, (iv) whole flaxseed supplementation significantly reduced insulin levels with the greatest effect for intervention duration of ≥ 12 weeks, and (v) flaxseed oil increased QUICKI, with the greatest effect for intervention duration of ≥ 12 weeks, mean age ≤ 50 years, and in patients with diabetes [68]. In a 2024 systematic review and meta-analysis involving 6 RCTs, Sabet and colleagues [29] showed a significant reduction in FBG levels (8.35 md/dL) following flaxseed intake for 10–24 weeks in patients with CAD.

Flaxseed supplementation significantly reduces FBG, insulin levels, and HOMA-IR, while increasing insulin sensitivity as measured by QUICKI, with no consistent effects on HbA1c. These benefits are more pronounced with whole flaxseed supplementation at doses ≥ 13 g/day, intervention durations ≥ 12 weeks, and in populations such as individuals with T2D, NAFLD, or baseline FBG ≥ 8 mmol/L or HbA1c ≥ 7.0%. The effects are particularly evident in participants with higher BMI (≥ 30 kg/m^2^) or younger age (≤ 50 years).

#### Liver markers

Although studies specifically targeting liver markers are limited, existing evidence suggests that flaxseed supplementation has been associated with decreased levels of ALT, AST, and GGT, markers of liver inflammation and damage, particularly in individuals with MASLD (NAFLD) [69–71].

Yari and colleagues [69] in 2016 conducted a two-arm open labeled RCT in 50 patients with NAFLD. Participants were assigned to take either a lifestyle modification program (LM), or LM + 30 g/day brown milled flaxseed for 12 weeks. At the end of the study, the liver enzymes -AST, ALT, and GGT—decreased significantly in both groups; however, this reduction was significantly greater in those who took flaxseed supplementation [69]. Ahmed and Fateh [56] in 2024 pooled the results of 5 RCTs in patients with NAFLD and showed significant reductions in levels of AST (21.18 mg/dl) and ALT (24.83 mg/dl) following flaxseed supplementation for 8–12 weeks. In 2024, Khodadadi and colleagues [71] conducted an open-label RCT in 100 patients with MASLD to investigate the effect of flaxseed supplementation and fasting mimicking diet (FMD) on surrogate measures of MASLD. Study participants were randomized to four groups: control group (lifestyle modification recommendations); flaxseed group (30 g/day of flaxseed powder consumption); FMD group (16 h of fasting per day); and combination of FMD with flaxseed. The results showed that serum liver enzymes (AST, ALT and GGT) decreased significantly in all intervention groups except for the control group [71].

#### Blood pressure

Evidence indicates that flaxseed supplementation can lower both SBP and DBP. Rodriguez-Leyva and colleagues [72] in 2013 conducted a RCT in which 110 participants with peripheral artery disease (PAD) were randomized to 30 g of milled flaxseed or placebo each day over 6 months. At the end of the study period, plasma levels of ALA and enterolignans increased 2- to 50-fold in the flaxseed-fed group but did not increase significantly in the placebo group. In the ground flaxseed group, SBP decreased by 10 mm Hg and DBP by 7 mm Hg compared with placebo after 6 months. Patients who entered the trial with a SBP ≥ 140 mm Hg at baseline experienced a significant reduction of 15 mm Hg in SBP and 7 mm Hg in DBP following flaxseed ingestion [72]. In a 2015 meta-analysis of 14 trials, Khalesi and colleagues [73] sought to clarify the effects of flaxseed supplementation on blood pressure and evaluated if this was influenced by baseline blood pressure, type of flaxseed supplementation, and duration of flaxseed supplementation. The results showed that flaxseed supplementation at doses ranging from 1.2–50 g/day for 3–48 weeks reduced SBP by 1.77 mmHg and DBP by 1.58 mm Hg. Though there was no significant evidence of effect modification by any of the subgroups evaluated, whole flaxseed significantly reduced DBP by 1.93 mm Hg and duration of flaxseed consumption ≥ 12 weeks also reduced DBP by 2.17 mmHg [73]. Ursoniu and colleagues [74] in 2016 pooled the results of 15 trials and reported significant reductions in both SBP (3 mmHg) and DBP (2 mmHg) following supplementation with flaxseed products at doses ranging from 1.2–60 g/day for 4 weeks to 12 months. In subgroup analyses, there was a greater effect on both SBP and DBP in trials with ≥ 12 weeks of duration, compared to trials lasting < 12 weeks. Regarding flaxseed supplement type, there was a significant reduction in SBP for flaxseed powder (1.81 mmHg), but not oil or lignan extract; DBP was significantly reduced with powder and oil preparations, 1.28 mmHg, and 4.10 mmHg, respectively, but not with lignan extract [74]. Mahmudiono and colleagues [75] in 2022 pooled the results of 5 RCTs to examine the effect of flaxseed oil consumption on blood pressure in patients with MetS and related disorders. Flaxseed consumption at doses ranging from 4–9.2 g/day for 6–12 weeks significantly reduced SBP (3.86 mmHg), with no effect on DBP [75]. In pooled analysis of 33 trials, Li and colleagues [76] in 2023 reported significant reductions in both SBP (3.19 mmHg) and DBP (2.61 mmHg) following flaxseed supplementation at doses ranging from 1–100 g/day for 3–48 weeks. Greater effects on SBP and DBP were found in trials with an intervention duration of > 20 weeks, ≥ 30 g/day of flaxseed, participants with BMI 25–30 kg/m^2^, and in patients with hypertension [76]. Xi and colleagues [46] in 2023 conducted a systematic review and meta-analysis of 13 RCTs to evaluate the impact of flaxseed supplementation on cardiometabolic parameters in patients with T2D. Their pooled analysis of 4 RCTs showed no effect of flaxseed supplementation on SBP or DBP [46]. In a 2024 systematic review and meta-analysis of 5 RCTs that aimed to evaluate the effectiveness of flaxseed supplementation on blood pressure in patients with hypertension, Fazeli Moghadam and colleagues [77] showed significant reductions in SBP (8.64 mmHg) and DBP (4.87 mmHg) following flaxseed supplementation at doses ranging from 500 mg/day-36 g/day for 8–24 weeks.

Evidence indicates that flaxseed supplementation can significantly reduce both SBP and DBP, with effects being more pronounced in certain subgroups. Studies show reductions in SBP ranging from 1.77 mmHg to 15 mmHg and DBP reductions from 1.28 mmHg to 7 mmHg, depending on dose, duration, and baseline characteristics. Greater effects were observed in interventions with higher doses (≥ 30 g/day), longer durations (≥ 12 weeks), and among participants with hypertension or elevated BMI.

### Hypertension

Although no studies have directly investigated the association between flaxseed supplementation and the incidence of hypertension, there is robust evidence supporting its role in blood pressure reduction among individuals with existing hypertension [72,77,78]. Several RCTs and meta-analyses have consistently demonstrated that flaxseed supplementation reduces both SBP and DBP [72,73,76,77]. In a notable study, daily consumption of 30 g of ground flaxseed for six months reduced SBP by 10 mmHg and DBP by 7 mmHg in hypertensive individuals [72]. Furthermore, some of the bioactive components of flaxseed such as lignans and fibre, have each been shown to be associated with a reduced risk of hypertension [79–82].

### Metabolic syndrome

No studies have directly examined the association between flaxseed supplementation and the overall risk of MetS. However, evidence from RCTs and meta-analyses suggests that flaxseed consumption exerts beneficial effects on several key parameters of MetS in patients with MetS and/or other comorbidities [70,83,84]. Flaxseed supplementation has been shown to significantly reduce both systolic and diastolic blood pressure [72,73,76,77], a critical component of MetS. Flaxseed improves insulin sensitivity and reduces insulin resistance, as measured by HOMA-IR scores, and stabilizes blood glucose levels [29,67,68]. The high fiber content of flaxseed promotes satiety and supports weight management, which may contribute to reductions in abdominal obesity over time. Flaxseed supplementation lowers TC, LDL-C, and TG levels [51], while improving HDL-C [54], addressing the dyslipidemia commonly associated with MetS.

### Metabolic dysfunction-associated steatotic liver disease (formerly NAFLD)

Although no studies have specifically examined the association between flaxseed supplementation and the risk of developing MASLD, evidence from RCTs supports its efficacy in managing the condition. Flaxseed supplementation has demonstrated several beneficial effects on metabolic parameters that are critical in MASLD management, such as improving lipid and liver enzyme profiles, reducing systemic inflammation, and enhancing insulin sensitivity. In the 2016 RCT by Yari and colleagues [69] in which 50 patients with NAFLD were randomized to LM or LM + 30 g/day brown milled flaxseed for 12 weeks, body weight, liver enzymes, insulin resistance and hepatic fibrosis and steatosis decreased significantly in both intervention groups at the end of the study period; however, the reductions were significantly greater in those who took flaxseed supplementation [69]. Yari and colleagues [70] again in 2021 conducted a RCT in which 100 patients with NAFLD were randomly assigned to four dietary intervention groups (LM (control) vs LM plus 30 g whole flaxseed powder vs LM plus 1 g hesperidin supplementation vs LM plus a combination of 30 g flaxseed and 1 g hesperidin). Following 12 weeks of dietary interventions, flaxseed or hesperidin supplementation or their combination significantly improved glucose and lipid metabolism, and reduced inflammation and hepatic steatosis [70]. Khodadadi and colleagues [71] in their open-label RCT published in 2024, investigated the effect of flaxseed supplementation and FMD on surrogate measures of MASLD using the following interventions: control group (lifestyle modification recommendations); flaxseed group (30 g/day of flaxseed powder consumption); FMD group (16 h of fasting per day); and combination of FMD with flaxseed. The results showed that serum TGs, TC, FBG and insulin, hs-CRP and liver enzymes decreased in all intervention groups. Hepatic steatosis and fibrosis scores were also decreased in the intervention groups [71].

While these findings suggest a therapeutic role for flaxseed in MASLD management, further research is needed to explore its preventive effects and direct impact on MASLD risk.

### Type 2 diabetes

While no studies have directly investigated the association between flaxseed supplementation and the risk of developing T2D, existing research demonstrates significant glycemic benefits in patients with T2D. Several RCTs and meta-analyses have shown that flaxseed supplementation can reduce FBG, insulin levels, and HbA1c, markers critical for diabetes management [29,67,68]. Additionally, flaxseed has been shown to lower insulin resistance, as indicated by improved HOMA-IR scores, and positively impact other glycemic measures, benefiting individuals with T2D or CHD [66,68]. These findings suggest that flaxseed may help improve glycemic control in those with T2D, potentially reducing disease progression and complications. There is also strong and consistent evidence linking each of flaxseed’s key bioactive components— ALA, lignans, and soluble fiber—with reduced risk of T2D [85–87].

### Chronic kidney disease

We identified no studies that have directly investigated the association between flaxseed supplementation and the risk of developing CKD. There is also no direct evidence to suggest that flaxseed supplementation can improve kidney function in CKD patients. However, flaxseed may exert beneficial effects on the kidney via its bioactive components. The lignans and omega-3 ALA in flaxseed have potent anti-inflammatory effects [88,89], which may mitigate inflammation-related kidney damage. Lignans in flaxseed can combat oxidative stress [89,90], which is linked to kidney damage. By reducing LDL-C and TGs, flaxseed may help lower cardiovascular risks commonly associated with CKD, potentially reducing the overall disease burden. Flaxseed’s blood pressure reducing effects are relevant for patients with CKD, as high blood pressure is a major contributor to kidney disease progression.

### Cardiovascular disease including CHD and stroke

Although no studies have directly investigated the associations between flaxseed supplementation and CVD endpoints, there is strong and consistent evidence linking each of its key bioactive components— ALA, lignans, and soluble fiber—to cardiovascular benefits. ALA is known to reduce inflammation, improve endothelial function, and lower blood pressure. Several individual studies as well as meta-analyses of these studies have consistently reported associations between increased ALA intake and reduced risk of CVDs including CHD and stroke [91–94].

The lignans in flaxseed have antioxidant and estrogenic properties [89], which may improve lipid metabolism, reduce oxidative stress, and modulate blood pressure, all of which support heart health. SDG is the major lignan found in flaxseed. There is consistence evidence linking higher intake of lignans with reduced risk of CVD [95].

Flaxseed’s soluble fiber helps lower TC and LDL-C by binding bile acids in the gut, thus reducing cholesterol absorption; soluble fibre also lowers blood pressure and reduces inflammation [38,96]. Just like ALA and lignans, there is a wealth of evidence linking higher dietary fibre intake with a reduced risk of cardiovascular outcomes [97,98].

### Other cardiovascular outcomes

#### Atrial fibrillation and arrhythmias

Direct studies on flaxseed’s effects on atrial fibrillation (AF) and arrhythmias are limited. Rodriguez-Leyva and colleagues [99] in their double-blinded RCT in 2019 evaluated whether daily consumption of a diet supplemented with 30 g of milled flaxseed vs placebo over 1 year by patients with PAD has effects on the prevalence of cardiac arrhythmias and exercise capacity. At baseline, patients had a high incidence of cardiac arrhythmias (48% in the flaxseed group and 32% in the placebo group). After 1 year, the presence of cardiac arrhythmias in the flaxseed group decreased by 2% and increased by 12% in the placebo group, though the difference was not statistically significant. The authors concluded that a larger sample size or a longer intervention with flaxseed may be required to show statistically significant differences [99]. There is also evidence showing that flaxseed may reduce the risk of these outcomes, primarily through its bioactive components [100].

### Heart failure

While flaxseed supplementation has not been directly studied in relation to heart failure, its bioactive components—ALA, lignans, and soluble fiber—have demonstrated mechanisms and effects that align with heart failure prevention and management strategies. There is evidence suggesting that higher dietary ALA intake is associated with a reduced risk of incident heart failure [101].

### All-cause mortality

Direct evidence linking flaxseed supplementation to all-cause mortality is limited. However, its potential to reduce all-cause mortality can be inferred from its impact on major cardiometabolic risk factors and outcomes. In the 2022 retrospective cohort analysis by Jiang and colleagues [45], patients who received flaxseed oil modestly had a better overall survival than those who received fish oil.

## Potential pathways underlying the beneficial cardiometabolic effects of dietary flaxseed and its bioactive components

**Fig. 1:**
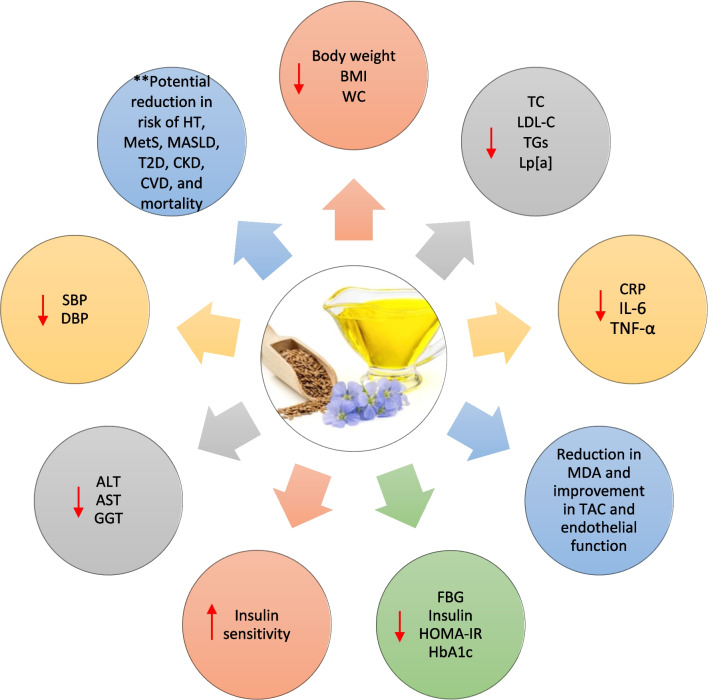
Dietary flaxseed and cardiometabolic risk factors and outcomes: summary of effects. ALT, alanine aminotransferase; AST, aspartate aminotransferase; BMI, body mass index; CKD, chronic kidney disease; CRP, C-reactive protein; CVD, cardiovascular disease; DBP, diastolic blood pressure; FBG, fasting blood glucose; GGT, gamma-glutamyltransferase; HT, hypertension; HOMA-IR, Homeostatic Model Assessment for Insulin Resistance; IL-6, interleukin-6; LDL-C, low-density lipoprotein cholesterol; Lp[a], lipoprotein[a]; MASLD, metabolic dysfunction-associated steatotic liver disease; MDA, malondialdehyde, MetS, metabolic syndrome; SBP. Systolic blood pressure; TAC, total antioxidant capacity; TC, total cholesterol; T2D, type 2 diabetes; TG, triglyceride; TNF-α, tumor necrosis factor-alpha; WC, waist circumference. **, Direct evidence on flaxseed’s impact on the risk of HT, MetS, MASLD, T2D, CKD, and CVD is lacking. However, its bioactive components—ALA, lignans, and soluble fiber—are known to be associated with reduced risks of these outcomes. Furthermore, flaxseed consumption exerts beneficial effects on several key parameters in patients with these conditions

The cardiometabolic benefits of dietary flaxseed are supported by its diverse bioactive components, which work through several pathways to improve cardiovascular health, metabolic balance, and overall cardiometabolic function. These key pathways include anti-inflammatory and antioxidant pathways, cholesterol-lowering mechanisms, blood pressure regulation, insulin sensitivity and glucose metabolism, gut microbiota modulation, endothelial function and vascular health, and weight management (Fig. 1). These pathways collectively contribute to reducing the risk of a spectrum of adverse cardiometabolic outcomes, including hypertension, MetS, MASLD, T2D, CKD, CVDs, and all-cause mortality.

Flaxseed contains high levels of ALA and lignans, particularly SDG, both of which have notable anti-inflammatory and antioxidant properties [88,90]. ALA is an omega-3 fatty acid that can reduce inflammation by lowering levels of pro-inflammatory cytokines like IL-6 and TNF-α [88]. SDG, a potent antioxidant, scavenges free radicals, reducing oxidative stress and protecting cells from damage [90], which is particularly beneficial in reducing the risk of atherosclerosis.

Flaxseed improves lipid profiles by reducing LDL-C and TC, partly due to its high fiber content and lignans. Soluble fiber in flaxseed binds bile acids, causing the liver to use up more cholesterol in producing bile acids, thus lowering blood cholesterol [96]. Additionally, lignans can inhibit cholesterol absorption and promote bile excretion, further aiding in cholesterol reduction [102,103].

Several bioactive components in flaxseed contribute to blood pressure regulation. The primary lignan in flaxseed, SDG, is metabolized by intestinal bacteria into enterolactone and enterodiol [35,36]. These metabolites can bind to estrogen receptors, mimicking or modulating the effects of endogenous estrogen.

The estrogen-like activity of flaxseed lignans may enhance vascular health by promoting vasodilation, reducing arterial stiffness, and exerting anti-inflammatory effects [37]. These actions can collectively improve blood flow and lower cardiovascular risk factors such as blood pressure. Additionally, flaxseed peptides (proteins) have been found to have angiotensin-converting enzyme (ACE) inhibitory activity, similar to some antihypertensive drugs, which helps to relax blood vessels and lower blood pressure [104]. ALA in flaxseed may inhibit soluble epoxide hydrolase, which alters oxylipin concentrations that contribute to the antihypertensive effects in patients with PAD [105,106]. Flaxseed’s demonstrated ability to lower both systolic and diastolic blood pressure may contribute to the prevention of AF and arrhythmias.

The soluble fiber in flaxseed forms a gel-like substance in the digestive tract, which slows the absorption of sugar into the bloodstream. This mechanism helps maintain stable blood sugar levels and can improve HbA1c levels, a marker of long-term blood sugar control [38]. Additionally, flaxseed may improve insulin sensitivity, likely due to the combined effects of its fiber, lignans, and ALA. This improvement in glucose metabolism reduces the risk of T2D and may benefit individuals already managing the condition [46]. Flaxseed’s high fiber content also aids in weight management; the soluble fiber in flaxseeds forms a gel-like substance in the digestive tract, slowing digestion and promoting a feeling of fullness.

Flaxseed acts as a prebiotic due to its fiber content, promoting the growth of beneficial gut bacteria. The fermentation of flaxseed fibers by gut microbiota produces short-chain fatty acids (SCFAs), which are known to improve gut health, reduce inflammation, and potentially enhance metabolic outcomes [107]. A healthier gut microbiota composition is also linked to better cardiometabolic health [108,109].

Flaxseed’s bioactive components, such as ALA and SDG, enhance endothelial function by promoting NO production [110,111], which is essential for blood vessel dilation and blood flow. Improved endothelial function reduces arterial stiffness and helps in preventing hypertension and vascular damage, both of which are critical for cardiovascular health. Improved endothelial function can also stabilize cardiac electrical activity, reducing the likelihood of arrhythmias [112].

In the body, ALA serves as a precursor to the longer-chain omega-3 fatty acids EPA and DHA, which play vital roles in cardiovascular and metabolic health [34]. Although the conversion of ALA to EPA and DHA is limited [34], EPA itself offers substantial cardioprotective effects. EPA is metabolized into eicosanoids, hormone-like substances that exhibit potent anti-inflammatory properties, which can reduce vascular inflammation, improve endothelial function, and modulate platelet aggregation [113,114]. The availability of ALA from flaxseed thus provides an essential source of omega-3 s for individuals who may not consume sufficient EPA and DHA from marine-based sources, offering a plant-based strategy for cardiometabolic health.

Collectively, these pathways highlight flaxseed’s role in reducing inflammation, managing blood lipids, controlling blood pressure, improving glucose metabolism, and supporting gut health. Through these mechanisms, flaxseed offers a holistic approach to managing and reducing cardiometabolic disease risk, making it an effective functional food for promoting cardiovascular and metabolic wellness.

## Impact of dietary flaxseed and its bioactive components on longevity and healthy aging

Longevity refers to the length of an individual’s life, often with a focus on extending lifespan, while healthy aging emphasizes maintaining physical, mental, and social well-being as one ages [115]. Healthy aging promotes resilience to age-related diseases and supports a high quality of life throughout the lifespan. Common strategies to enhance longevity and promote healthy aging include balanced diets rich in whole foods, caloric restriction, regular physical activity, stress reduction, and sleep hygiene.

Dietary flaxseed has emerged as a functional food with the potential to support longevity and healthy aging, owing to its diverse cardiometabolic benefits. Its bioactive components—including omega-3 fatty acids (ALA), lignans, and fiber—improve cardiovascular health by lowering blood pressure, enhancing lipid profiles, and reducing systemic inflammation. These effects are essential for preventing chronic conditions such as CVD and T2D, which are leading causes of morbidity and mortality in older adults. By addressing these risk factors, flaxseed may contribute to an extended health span and promote longevity.

Cardiometabolic conditions such as CVD, T2D, and MetS are age-related diseases, with their incidence rising significantly as individuals age. The biological mechanisms driving the aging process—including chronic inflammation, oxidative stress, and metabolic dysregulation—are central to the pathogenesis of these conditions [116]. Flaxseed, with its rich bioactive components such as ALA, lignans, and soluble fiber, impacts several critical biological pathways to promote healthy aging.

Aging is associated with a chronic, low-grade inflammatory state, often termed “inflammaging,” which plays a pivotal role in age-related cardiometabolic conditions [116–124]. Flaxseed’s ALA is a precursor to anti-inflammatory eicosanoids that downregulate inflammation, reducing markers such as CRP [113,114]. Lignans such as SDG modulate inflammatory pathways by inhibiting the production of pro-inflammatory cytokines like IL-6 and TNF-α [125].

Oxidative stress, caused by an imbalance between ROS and antioxidant defenses, accelerates cellular aging and damages lipids, proteins, and DNA [118,126–132]. Lignans and ALA neutralize ROS, protecting cellular components from oxidative damage [88,90,133]. By reducing oxidative stress, flaxseed lowers MDA levels, a marker of lipid peroxidation. These antioxidant effects are integral to slowing the aging process and reducing the risk of cardiometabolic diseases.

Insulin resistance and impaired glucose metabolism are hallmarks of aging and key drivers of T2D [134,135]. Flaxseed enhances insulin sensitivity and regulates blood glucose levels. Soluble fiber in flaxseed slows glucose absorption, stabilizing blood sugar levels [38]. Lignans improve insulin sensitivity by modulating glucose metabolism and reducing hyperglycemia-induced cellular stress [136,137]. These effects prevent metabolic dysregulation, a common issue in aging populations [138].

The gut microbiome undergoes significant changes with age, often resulting in dysbiosis, which is linked to inflammation and metabolic disorders [139,140]. Flaxseed’s fiber content promotes a healthy gut microbiome; soluble fiber serves as a prebiotic, enhancing the growth of beneficial gut bacteria such as Bifidobacteria and Lactobacilli [107]. Lignans are metabolized by gut bacteria into enterolignans, which exert systemic health benefits [35,36]. A balanced gut microbiome improves intestinal barrier function, reduces inflammation, and enhances metabolic health, contributing to healthy aging.

Mitochondrial dysfunction is a hallmark of aging, leading to reduced energy production and increased oxidative stress [141,142]. Flaxseed’s bioactive components support mitochondrial health. ALA enhances mitochondrial membrane integrity and reduces mitochondrial ROS production [143]. By improving mitochondrial function, flaxseed promotes cellular energy balance and reduces age-related cellular damage.

DNA damage accumulates with age, contributing to genomic instability and cellular senescence [144,145]. Lignans exhibit protective effects against DNA damage induced by oxidative stress [146]. Some of the bioactive components of flaxseed might be associated with reduced telomere shortening [147,148], a marker of cellular aging, though more research is needed in this area.

Sirtuin-1 (SIRT1), a protein critical for longevity, regulates cellular processes such as stress resistance, metabolism, and DNA repair [149]. Lignans and ALA indirectly promote SIRT1 activity by reducing oxidative stress and improving metabolic homeostasis [150,151]. Enhanced SIRT1 activity has been linked to delayed aging and reduced risk of cardiometabolic diseases [152,153].

Autophagy, the process by which cells remove damaged components, declines with age, contributing to cellular dysfunction [154,155]. ALA and lignans improve cellular stress responses, indirectly promoting autophagy [156,157]. Enhanced autophagy helps clear damaged mitochondria and proteins, improving cellular health and longevity.

In summary, dietary flaxseed and its bioactive components exert profound effects on several biological processes central to healthy aging. By addressing key mechanisms such as inflammation, oxidative stress, insulin resistance, mitochondrial dysfunction, and impaired autophagy, flaxseed supports the prevention of age-related cardiometabolic conditions. Its ability to influence gut health, DNA repair, telomere maintenance, and SIRT1 activity further underscores its potential to enhance longevity and promote healthy aging. Future research should explore these pathways in greater detail to fully harness the potential of flaxseed in aging interventions.

## Adverse effects of dietary flaxseed

Dietary flaxseed is generally considered safe when consumed in moderate amounts; however, some individuals may experience adverse effects. Common gastrointestinal symptoms include bloating, gas, stomach pain, and diarrhea, which are mainly due to its high fiber content. At higher doses, these symptoms may intensify, leading to discomfort and digestive issues. In rare cases, flaxseed, particularly its oil, can interfere with blood clotting, especially when taken with anticoagulant medications, due to the potential of ALA to reduce platelet aggregation [158]. Additionally, flaxseed contains small amounts of cyanogenic glycosides, compounds that can release cyanide in high amounts [159]. While this is not typically an issue with standard dietary intake, excessive consumption should be avoided. It’s important to note that flaxseed oil contains high levels of phosphorus [160], a mineral that individuals with CKD often need to limit. Excessive phosphorus intake can lead to complications in CKD patients, such as bone and cardiovascular issues. Therefore, CKD patients should exercise caution with flaxseed oil supplementation and consult their healthcare providers before making any dietary changes. For individuals with hormone-sensitive conditions, caution may be warranted since flaxseed’s lignans have mild estrogen-like effects [37]. Pregnant and breastfeeding women, as well as individuals on hormone therapies, should consult healthcare providers before using flaxseed in large amounts. These potential risks have been noted in the context of short-term use. The long-term implications of flaxseed supplementation, particularly concerning potential risks, are an area with limited evidence.

## Clinical and public health implications

The findings on flaxseed supplementation underscore its potential as a valuable dietary intervention for improving cardiometabolic health, enhancing longevity, and promoting healthy aging. Flaxseed supplementation has demonstrated modest but meaningful reductions in anthropometric indices such as body weight, BMI, and WC, particularly with ground whole flaxseed at doses of ≥ 30 g/day, intervention durations of at least 12 weeks, and among individuals with higher baseline BMI (≥ 27 kg/m^2^ or > 30 kg/m^2^). These findings suggest that flaxseed can serve as an adjunct to weight management strategies, particularly for individuals at heightened risk of obesity-related complications. In terms of lipid profiles, flaxseed supplementation significantly reduces TC, LDL-C, TGs, and Lp[a], with more pronounced effects observed for ground whole flaxseed, higher doses, and longer durations of supplementation. These reductions highlight flaxseed’s role in dyslipidemia management, a key strategy in preventing CVDs. Its ability to lower markers of inflammation such as hsCRP and IL-6, reduce oxidative stress markers like MDA, and improve TAC further emphasizes its utility in mitigating chronic low-grade inflammation and oxidative stress—both of which are central to cardiometabolic diseases and aging. Flaxseed’s benefits extend to glycemic control, as it significantly lowers FBG, insulin levels, and HOMA-IR while increasing insulin sensitivity (QUICKI). These effects are particularly evident with ground whole flaxseed supplementation in populations with T2D, NAFLD, or elevated baseline glycemic markers. Improvements in circulating liver markers, such as ALT, AST and GGT, and reductions in blood pressure add to flaxseed’s broad cardiometabolic benefits, suggesting its applicability for individuals with MASLD, hypertension, and other related conditions. Although no RCTs have directly assessed the impact of flaxseed supplementation on the risk of hypertension, MetS, T2D, CKD, or CVDs, the consistent evidence linking its individual bioactive components—ALA, lignans, and soluble fiber—to reductions in relevant risk factors as well as the risk of these conditions, suggests a protective role. Whether there are sex differences in the cardiometabolic effects of flaxseed supplementation are unclear. The current findings highlight the potential for flaxseed to be incorporated into public health strategies aimed at preventing these conditions.

### Recommendations

Flaxseed is recognized for its potential cardiometabolic and health benefits, but the lack of universal dosage guidelines and standardization of supplementation makes it challenging to provide specific recommendations for the general population. In 2014, Health Canada endorsed a health claim for ground whole flaxseed based on a meta-analysis of 8 RCTs, which demonstrated significant reductions in TC and LDL-C with ground flaxseed supplementation [50]. The approved claim states that a daily intake of 40 g (approximately 5 tablespoons) of ground flaxseed can help lower cholesterol [50]. This recommendation is particularly relevant for the Canadian population, where high cholesterol levels are prevalent [161]. While Dietary Guidelines of Canada and other countries do not provide specific portion recommendations for flaxseed consumption, 1–2 tablespoons per day is generally recommended. One tablespoon (7 g) of ground flaxseed contains 37 cal and 2 g of polyunsaturated fat, including omega-3 fatty acids, 0.5 g of monounsaturated fat and 2 g of dietary fiber.

The findings from this review underscore the importance of considering the type or form, dose, and duration of flaxseed supplementation for achieving optimal cardiometabolic outcomes. Despite the inconsistencies in the literature, the overall evidence suggests that a dose of ≥ 30 g/day of ground whole flaxseed, taken for a minimum of 12 weeks, yields the most consistent and comprehensive benefits in improving lipid and blood pressure levels, improving measures of glycemia, and lowering inflammation. These findings suggest that individuals seeking cardiovascular benefits from flaxseed should prioritize whole or ground flaxseed rather than flaxseed oil, as flaxseed oil, while rich in ALA, lacks the fiber and lignans found in the whole seeds, which are key to many of flaxseed’s health benefits. Therefore, the choice of flaxseed supplementation should align with the individual’s specific health objectives, whether they are focused on lowering cholesterol, managing weight, or enhancing metabolic health.

Flaxseed’s ability to influence key biological processes such as inflammation, oxidative stress, autophagy, and telomere maintenance further aligns it with the goals of healthy aging and longevity. These mechanisms play a central role in delaying the onset of age-related diseases and extending health span. Given these promising findings, healthcare providers and public health practitioners should consider recommending flaxseed as part of a balanced diet, especially for individuals at risk of cardiometabolic diseases or those seeking to promote healthy aging. This can be a particularly valuable dietary addition for individuals at risk of conditions like MASLD, T2D, or CVD. Flaxseed could support healthy aging by targeting age-related inflammation, oxidative stress, and insulin resistance. Regular flaxseed consumption may also benefit gut health, which often deteriorates with age. Flaxseed’s blood pressure-lowering effects make it a practical dietary intervention for reducing systolic and diastolic blood pressure when integrated with broader hypertension management strategies. Flaxseed’s ability to stabilize blood glucose levels and enhance insulin sensitivity positions it as a functional food for managing patients with MetS or T2D while simultaneously addressing other cardiometabolic risk factors like obesity and dyslipidemia.

While flaxseed offers substantial health benefits, variability in individual health needs, supplement forms, and dosages means that it is advisable for individuals to consult with healthcare providers or registered dietitians before incorporating flaxseed supplements into their routine. Personalized recommendations based on an individual’s health status, goals, and other medications or conditions will help optimize flaxseed’s benefits while avoiding potential adverse effects or interactions. For example, recommending flaxseed as a breakfast addition (e.g., mixed into oatmeal, smoothies, or baked goods) provides an easy and practical way to encourage regular consumption.

At a broader level, flaxseed could be integrated into national dietary guidelines to address public health priorities, particularly the growing prevalence of obesity, MetS, and CVD. Public health campaigns could emphasize the benefits of incorporating plant-based functional foods like flaxseed into everyday diets, with tailored messaging for high-risk populations such as individuals with MASLD, T2D, or CVD. To enhance the practical integration of flaxseed into clinical and public health strategies, future research should focus on long-term RCTs to better understand flaxseed’s effects on hard clinical outcomes, refine dosage guidelines, and explore its potential in enhancing longevity and promoting healthy aging on a broader scale. Such studies will be essential in establishing more precise recommendations and validating the therapeutic potential of flaxseed for both cardiometabolic health and age-related conditions.

### Comparative benefits of flaxseed among functional foods

Flaxseed stands out among functional foods due to its unique combination of bioactive components, including ALA, lignans, and dietary fiber. While other plant-based foods, such as chia seeds [162–165], walnuts [166–169], and oats [170–178], offer benefits for cardiometabolic health, flaxseed provides a broader spectrum of effects by addressing multiple pathways simultaneously. For example, walnuts are a rich source of omega-3 fatty acids [179] but lack the lignans found in flaxseed, which are critical for modulating inflammation and supporting hormonal balance. Similarly, oats are effective in lowering cholesterol due to their beta-glucan content [180] but do not offer the same antioxidant or anti-inflammatory properties as flaxseed. Chia seeds share similarities with flaxseed in terms of fiber and omega-3 content [181] but have a less established evidence base regarding blood pressure reduction and glycemic control. These distinctions highlight flaxseed’s unique potential not only as a cardiometabolic intervention but also as a food with broader implications for healthy aging and longevity. Its ability to target processes like inflammation, oxidative stress, and insulin resistance, coupled with its role in supporting gut health and mitochondrial function, underscores its value in dietary strategies for both disease prevention and healthspan extension.

### How to incorporate flaxseed into the diet

Flaxseed is versatile and can be easily integrated into a variety of meals, making it an accessible functional food for individuals aiming to improve cardiometabolic health or support healthy aging [182,183]. Ground flaxseed is the preferred form for consumption, as it allows for better nutrient absorption compared to whole flaxseed, which often passes undigested through the digestive tract. Flaxseed can be added to breakfast items like oatmeal, smoothies, or yogurt, providing a simple and effective way to include its beneficial components in the daily diet. It can also be incorporated into baked goods such as muffins, pancakes, or bread, replacing some of the flour or fat content. For savory dishes, flaxseed can be sprinkled over salads, soups, or roasted vegetables. Additionally, flaxseed can serve as a vegan egg substitute in recipes, where one tablespoon of ground flaxseed mixed with three tablespoons of water acts as a binding agent. Flaxseed oil, while rich in ALA, lacks the lignans and fiber found in whole or ground flaxseed. It is best used as a salad dressing or finishing oil to avoid heat degradation of its omega-3 content, rather than for cooking. For individuals new to flaxseed, starting with small amounts (e.g., 1–2 tablespoons per day) is advisable to minimize potential digestive discomfort, gradually increasing intake as tolerated.

Cultural dietary patterns play a pivotal role in shaping food preferences, preparation methods, and consumption habits, which in turn can affect the integration of flaxseed into diets. In regions where flaxseed is not a traditional food item, its unfamiliarity may present challenges in acceptance and utilization. However, these barriers can be mitigated through tailored public health campaigns and culinary education that emphasize the nutritional benefits of flaxseed and provide culturally appropriate preparation methods. For instance, flaxseed can be incorporated into traditional dishes by grinding it into flour for baking, sprinkling it on staple foods, or blending it into beverages. Additionally, collaborations with local food producers and chefs can help create culturally adapted recipes that incorporate flaxseed seamlessly. Strategies like these have been successful in promoting other functional foods globally. Addressing cultural preferences and dietary habits is very essential for the widespread adoption of flaxseed, particularly in low- and middle-income countries where cardiometabolic diseases are on the increase. These considerations should be integral to future implementation strategies aimed at leveraging flaxseed’s health benefits.

## Gaps in the evidence and future directions

Despite promising findings on the benefits of flaxseed supplementation for cardiometabolic health, several gaps in the evidence remain, emphasizing the need for further research. While current research overwhelmingly supports the benefits of flaxseed supplementation on cardiometabolic risk factors, most studies to date have been conducted in relatively small populations and over short-to-medium term durations, limiting the ability to assess long-term effects and any cumulative risks. Potential risks, such as gastrointestinal discomfort, cyanogenic glycoside content, or interactions with medications, have been noted in the context of short-term use but require further exploration for long-term supplementation. For instance, cyanogenic glycosides in flaxseed are generally rendered harmless through processing, but their safety with chronic high-dose use remains understudied. To address these gaps, larger RCTs with extended durations are essential. These studies should aim to evaluate not only the durability of cardiometabolic benefits but also any unintended consequences associated with prolonged supplementation.

There is also a paucity of studies examining the effects of flaxseed supplementation on hard clinical outcomes such as MetS, MASLD, T2D, CKD, and CVD. While long-term trials would be ideal, observational cohort studies could help fill this gap by exploring associations between flaxseed consumption and these outcomes in real-world settings. Such studies could provide critical insights into the specific doses, forms, and frequencies of flaxseed intake that optimize benefits across diverse populations.

Inconsistencies in the literature concerning the forms of flaxseed supplementation and intervention durations are notable and merit closer examination. The studies reviewed included various forms of flaxseed, such as whole flaxseed, ground flaxseed (flax meal), flaxseed oil, and lignan extracts, each of which differs in its nutritional composition and potential health effects. These differences can lead to variability in the reported benefits, particularly for outcomes such as lipid profiles, glycemic control, and markers of inflammation. Intervention duration also plays a critical role. The evidence suggests that a dose of ≥ 30 g/day of ground whole flaxseed for a minimum of 12 weeks consistently yields the most comprehensive cardiometabolic benefits. Shorter interventions or lower doses, while still beneficial in some contexts, often produce less pronounced effects or fail to reach statistical significance. Furthermore, whole flaxseed and ground flaxseed generally demonstrate more consistent benefits compared to flaxseed oil or lignan extracts, particularly for improving lipid profiles and inflammatory markers. Addressing these inconsistencies underscores the need for standardization in future research. Future studies should aim to specify the form, dose, and duration of flaxseed supplementation while ensuring sufficient follow-up periods to capture meaningful outcomes.

Tailoring flaxseed recommendations to different populations is challenging due to limited subgroup analyses and a lack of studies in diverse demographic groups. Factors such as age, sex, baseline health status, cultural dietary patterns, and genetic predispositions may influence the efficacy of flaxseed supplementation. For broader applicability, future research should prioritize well-powered studies in diverse populations to elucidate the variability in response to flaxseed. Such studies could refine dosing recommendations and expand the utility of flaxseed as a functional food across different population groups and cultural contexts. This approach would ensure that dietary recommendations are both evidence-based and inclusive.

Mechanistic studies are critical for deepening our understanding of the biological pathways through which flaxseed exerts its benefits. Beyond its well-established anti-inflammatory, antioxidant, and metabolic effects, the gut microbiome represents a particularly promising frontier. Dysbiosis, a common feature of aging, is linked to chronic inflammation, insulin resistance, and metabolic disorders [184]. Flaxseed’s fiber content acts as a prebiotic, fostering the growth of beneficial bacteria and enhancing the production of bioactive metabolites such as enterolignans. These metabolites may play significant roles in systemic health by modulating inflammation, improving glucose metabolism, and enhancing gut barrier integrity. Future research should explore how flaxseed supplementation can counteract age-related dysbiosis and its downstream effects, including chronic inflammation, metabolic dysfunction, and cardiometabolic risk.

Research should also prioritize flaxseed’s potential to influence aging-related pathways. The effects of ALA and lignans on mitochondrial function, DNA repair, and telomere maintenance remain underexplored. Investigating how flaxseed impacts processes such as oxidative stress reduction, autophagy enhancement, and SIRT1 activation could provide valuable insights into its role in promoting healthy aging and extending healthspan. Furthermore, studies focusing on flaxseed’s ability to improve resilience to cellular and molecular damage could clarify its contribution to longevity.

Future research should prioritize direct comparative studies between flaxseed and other functional foods to better delineate its unique benefits for cardiometabolic health and healthy aging. Functional foods such as chia seeds, walnuts, quinoa, soy, and oats have all been shown to possess health-promoting properties, including anti-inflammatory, antioxidant, and lipid-lowering effects [179–181,185]. Direct comparisons could evaluate how flaxseed differs or complements these foods in terms of its bioactive components, such as ALA, lignans, and dietary fiber. For example, comparative studies could assess the efficacy of flaxseed versus chia seeds or walnuts, both of which are rich in omega-3 fatty acids, in reducing inflammation and improving lipid profiles. Similarly, flaxseed could be compared with oats, which are known for their beta-glucan content [180], in terms of cholesterol-lowering effects and glycemic control. Such research would not only provide a clearer understanding of flaxseed’s unique contributions but also inform dietary recommendations that combine various functional foods for synergistic health benefits. These studies should also explore outcomes related to longevity and resilience against age-related diseases, such as CVD, T2D, and MetS. RCTs with robust designs, including crossover studies, would be ideal to provide high-quality evidence on the comparative effectiveness of these functional foods.

Few studies have compared different flaxseed preparations—such as whole seeds, ground seeds, and oil—and their relative efficacy in addressing specific health outcomes. Understanding these differences could guide more precise dietary recommendations.

The role of sex differences in the cardiometabolic effects of flaxseed supplementation is an important area of inquiry, but it remains underexplored. Biological differences between men and women, including variations in hormone levels, metabolism, and body composition, could potentially influence the response to flaxseed’s bioactive components. Preliminary evidence suggests that lignans, which are phytoestrogens, may have different effects based on hormonal status, potentially providing greater benefits in postmenopausal women due to their estrogenic activity effects [37]. Similarly, sex-specific differences in lipid metabolism may modulate the impact of flaxseed on cholesterol levels. Future research should prioritize the investigation of sex-specific and age-specific responses to flaxseed supplementation to provide more tailored dietary recommendations. RCTs stratified by sex or secondary analyses of existing data could shed light on whether men and women derive differing cardiometabolic benefits from flaxseed.

Emerging technologies, such as multi-omics approaches (e.g., metabolomics, transcriptomics, and microbiome sequencing) and advanced imaging techniques, offer new opportunities to uncover the broader impacts of flaxseed on systemic health. These tools could help elucidate the interplay between flaxseed’s bioactive components and key biological systems, providing a foundation for precision nutrition strategies.

Addressing these evidence gaps is critical for refining dietary recommendations and integrating flaxseed into public health strategies aimed at preventing cardiometabolic diseases and promoting healthy aging. By leveraging the potential of flaxseed, future research can inform evidence-based interventions that not only mitigate the global burden of cardiometabolic diseases but also enhance longevity and overall quality of life.

## Conclusions

The evidence highlights flaxseed supplementation as a promising dietary strategy for improving cardiometabolic health and addressing key public health challenges. Flaxseed has demonstrated substantial benefits across a range of cardiometabolic risk factors, including reductions in body weight, BMI, waist circumference, blood lipids (TC, LDL-C, TGs and Lp[a]), markers of inflammation and oxidative stress, fasting blood glucose, and insulin resistance. Additionally, its effects on blood pressure and liver health underscore its broad therapeutic potential. These benefits are most pronounced with ground whole flaxseed at doses of ≥ 30 g/day and supplementation durations of at least 12 weeks, particularly among individuals at high cardiometabolic risk such as those with elevated BMI, hypertension, T2D, or other metabolic disorders.

Flaxseed’s bioactive components—ALA, lignans, and soluble fiber—play a central role in these benefits, contributing not only to improved cardiometabolic markers but also to systemic health improvements. By targeting key biological processes such as inflammation, oxidative stress, and metabolic dysregulation, flaxseed aligns with pathways involved in healthy aging and longevity. This positions flaxseed as a valuable dietary intervention to promote long-term health, delay the onset of age-related diseases, and enhance quality of life.

Despite its potential, significant gaps in the evidence remain. Many existing trials have been short-term with small sample sizes, limiting their ability to evaluate the long-term effects of flaxseed supplementation on hard outcomes such as MetS, MASLD, CKD, T2D, and CVDs. Large-scale, long-term RCTs and observational studies are needed to confirm these associations and establish the sustained benefits of flaxseed on these outcomes. Further research should also aim to refine the optimal dose, type, and frequency of flaxseed supplementation to maximize its benefits across diverse populations. Mechanistic studies are equally important to better understand the pathways underlying flaxseed’s broad effects, particularly in the context of healthy aging and longevity. Integrating flaxseed supplementation into dietary guidelines and public health strategies could offer a cost-effective and scalable solution to the growing global burden of cardiometabolic diseases. As evidence continues to build, flaxseed has the potential to become a cornerstone of dietary recommendations for cardiometabolic health and age-related disease prevention. Future research should focus on closing existing knowledge gaps and exploring flaxseed’s role in promoting healthy aging, ultimately providing a robust foundation for evidence-based recommendations in both clinical and public health practice.

## Data Availability

This is a narrative review; no new scientific data was generated, and all data are within the paper.
